# CD24: A Rheostat That Modulates Cell Surface Receptor Signaling of Diverse Receptors

**DOI:** 10.3389/fcell.2016.00146

**Published:** 2016-12-27

**Authors:** D. Craig Ayre, Sherri L. Christian

**Affiliations:** Department of Biochemistry, Memorial University of NewfoundlandSt. John's, NL, Canada

**Keywords:** CD24, signal transduction, protein interaction, cellular activation, surface receptor, GPI-anchored protein, extracellular stress signaling, receptor partner

## Background

CD24 (also called Heat Stable Antigen or nectadrin) is expressed on many cell types (Fang et al., 2010), and its expression is dynamically regulated during cellular differentiation (Ayre et al., 2016). CD24 was first described for its role in blocking B lymphocyte (B cell) development. Transgenic mice overexpressing CD24 and CD24 knockout mice exhibit a loss of immature B cells via their increased apoptosis (Hough et al., 1996; Nielsen et al., 1997) demonstrating that altering CD24 expression has serious repercussions in these cells. Additionally, antibody (Ab)-mediated engagement of CD24 induces apoptosis or suppresses proliferation of B cells, depending on their developmental stage (Chappel et al., 1996). CD24 also mediates homeostatic proliferation in T cells (Li et al., 2004), and can negatively regulate inflammation by inhibiting dendritic cell (DC) activation (Chen et al., 2009).

CD24 also acts outside the immune system. It supports the differentiation of immature pre-adipocytes into adipocytes (Rodeheffer et al., 2008; Fairbridge et al., 2015; Smith et al., 2015) and is a positive regulator of cerebellar neurite outgrowth, but a negative regulator for dorsal root ganglion neurogenesis (Kleene et al., 2001). In contrast, CD24 negatively regulates corneal growth to inhibit the development of pterygium (Riau et al., 2011). CD24 can regulate both pro- and anti-proliferative effects in cancer cells, and both increase or reduce metastasis, depending on the cancer type (Kristiansen et al., 2004; Ju et al., 2011). Finally, we and others have identified CD24 as being carried on exosomes and microvesicles (Keller et al., 2007; Ayre et al., 2015; Grigor'eva et al., 2016), where its ultimate function is unknown.

CD24 has also been implicated in regulating cell stress. In a model of acetaminophen-induced liver damage, wild type mice regulated liver inflammation via CD24 and Siglec-G on DCs, whereas in CD24 knockout mice, the same challenge proved fatal (Chen et al., 2009). In addition, hypoxia in solid tumors induced CD24 expression via Hypoxia Inducible Factor-1 leading to increased tumor cell survival (Thomas et al., 2012). Finally, CD24 has been shown to regulate angiogenesis through Heat Shock Protein 90 and modulation of the STAT3/VEGF pathway (Wang et al., 2016). Therefore, CD24 is also important for modulating the sensitivity or cellular response to extracellular stresses.

CD24 is a glycophosphatidylinositol (GPI)-anchored protein, possessing no intracellular signaling domains (Kay et al., 1991). However, several signal transduction proteins are associated with CD24 activity. The best-described are the Src-family protein tyrosine kinases Lyn, Fyn, Fgr, Lck and Hck but how these are activated is unknown (Sammar et al., 1997; Suzuki et al., 2001; Fang et al., 2010; Su et al., 2012). Many ligands have been identified for CD24 including P-, L-, and E- Selectin, High Mobility Group Box 1 (HMGB1), L1 cell adhesion molecule (L1CAM), Neural cell adhesion molecule (NCAM1) and Siglec-G (Aigner et al., 1995; Myung et al., 2011; Tan et al., 2016). To date, no mechanism has been proposed that explains the contradictory nature of the processes regulated by CD24, its apparent lack of intrinsic signaling capability, or its diverse collection of reported ligands.

## CD24 as a signaling rheostat

**Figure 1:**
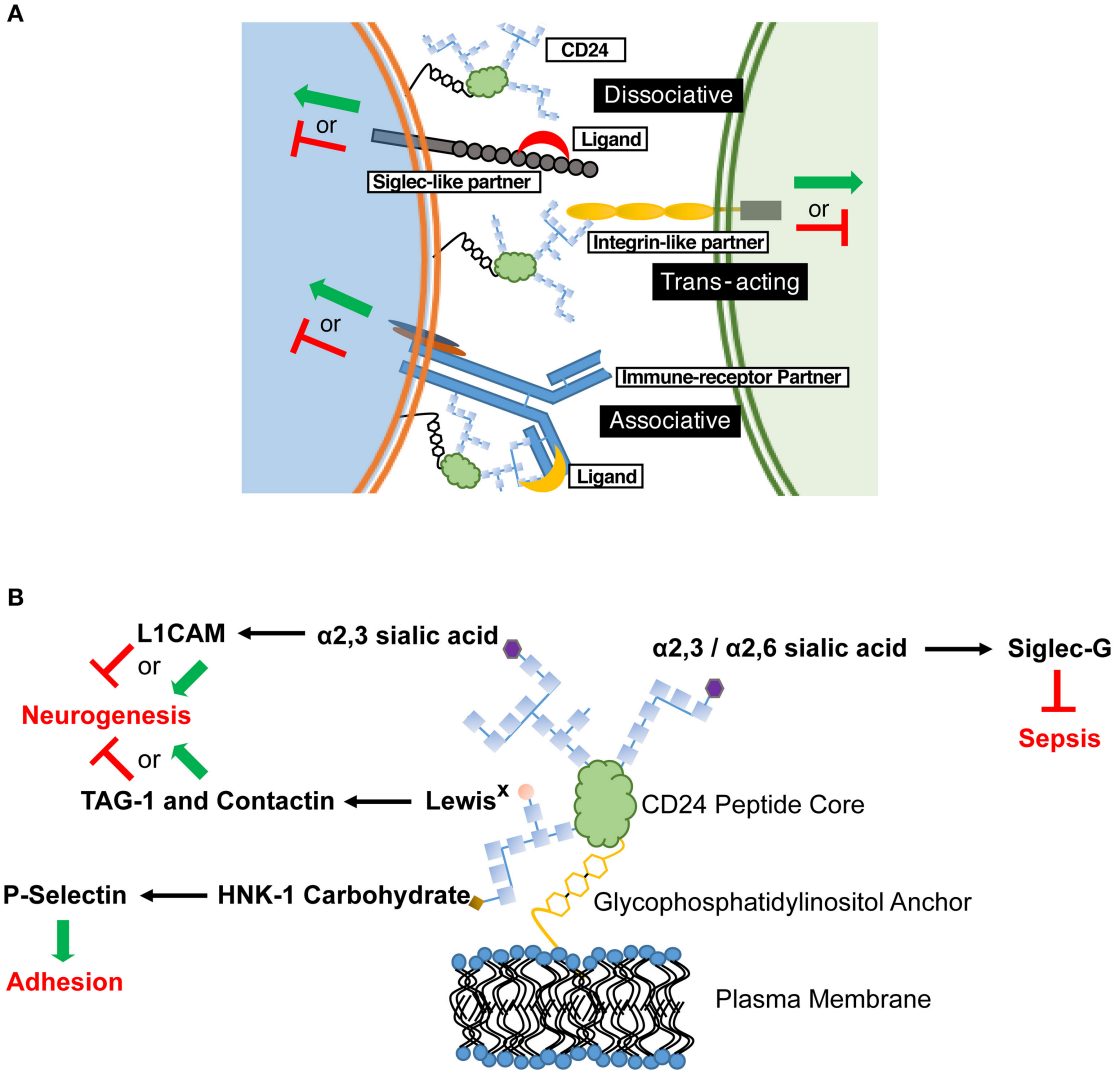
**CD24 operates through a combination of ***in cis*** and ***in trans*** partners to affect cell behavior in a cell-specific manner. (A)** Schematic diagram showing the possible associations of CD24 with partner receptors and ligands. These associations selectively tune cellular responses. CD24 association with a signaling partner may be enhanced or inhibited through associative and dissociative ligands, respectively. The various CD24 interactions may not be mutually exclusive on a single cell, thus leading to a mosaic of cellular interactions and activation (green arrow) or inhibitory (red line) effects. **(B)** Interactions between specific CD24 glycosylations, ligands and known biological outcomes. Glycosylations are depicted as chains of carbohydrate monomers (blue squares) but do not represent a specific structure. The ligand-interacting, terminal carbohydrate moiety is indicated. L1CAM, Contactin and TAG-1 show both activating and inhibitory signals for neurogenesis as both effects can be mediated in discrete regions during CNS development.

Our opinion is that CD24 functions as a rheostat to modulate responses transduced by partnered cell surface receptor(s), and that these partners define the biological outcomes observed. Mechanistically, CD24 likely modulates activation of this partner through direct physical interaction mediated by its modifiable glycoslyations. The variable nature of CD24-mediated effects can thus be explained by its *in cis* association with unique, cell-type specific signaling partners (Figure 1A). The activity of the partner receptor could be further modulated through additional *cis* or *trans* elements acting via multi-receptor complexes. Moreover, the activity of the partner receptor could affect other downstream receptors. The presence or absence of CD24 may also alter the association of cell surface receptors with their canonical ligands, to promote or inhibit receptor activation. Similarly, direct ligation of CD24 could also affect its association and modulation of a partnered receptor. These ligand interactions could also promote association, or displacement, of CD24 from its receptor partner, which we term an associative or dissociative ligand, respectively.

While it is our opinion that this is the most logical explanation for the cell-specific effects mediated by CD24, it does not suggest a generalized mechanism for all GPI-anchored proteins. Others have been shown to work through specific transmembrane proteins, via endocytosis, or through lipid kinases (Kamen et al., 1988; Deckert et al., 1996; Stulnig et al., 1997; Horejsí et al., 1998; Suzuki et al., 2007; Paulick and Bertozzi, 2008), thus it is necessary to evaluate the mechanism of signaling for each GPI-anchored protein individually. Importantly, we believe that CD24 is unique in that it partners with different and, specific signaling receptors in a cell-type dependent manner.

### Supporting rationale

#### Physical interactions with cell surface receptors

CD24 interacts *in cis* with L1CAM on neuroblastoma cells in a predicted 5:1 ratio (Kadmon et al., 1995). L1CAM/CD24 complexes also associate *in cis* with NCAM1, forming a tri-molecular complex, however no direct interaction between CD24 and NCAM1 was observed. The use of Ab against CD24 or L1CAM, to mimic ligand, induced a cellular calcium influx, with co-stimulation having a synergistic effect (Kadmon et al., 1995). This strongly suggests that the physical interaction between CD24 and L1CAM is associated with shared signaling processes.

CD24 also acts *in cis* with Siglec-G to moderate DC activation (Chen et al., 2009, 2011). In DC from the liver, CD24 forms a complex between Siglec-G and extracellular danger-associated molecular pattern (DAMP) proteins, such as HMGB1, to alter Toll-Like Receptor (TLR) activity (Chen et al., 2009; Liu et al., 2009). In the presence of CD24, Siglec-G inhibits the activation of TLRs by DAMPS. However, in the absence of CD24, the inhibition of TLR is lost. The interaction between CD24 and Siglec-G is facilitated by the glycosylations on CD24. Moreover, CD24 is a necessary mediator in this system as Siglec-G and HMGB1 are associative ligands of CD24, but neither HMGB1 nor TLR directly interacted with Siglec-G in this system (Chen et al., 2009).

#### Other interactions with signaling proteins and receptors

Studies in B cells showed that CD24 alters the localization of the B Cell Receptor (BCR) and associated intracellular signaling proteins within lipid rafts (Suzuki et al., 2001). Furthermore, engagement of the BCR or CD24 results in many of the same outcomes, including apoptosis, Protein Tyrosine Kinase (PTK) and Mitogen Activated Protein Kinase (MAPK) activity (Suzuki et al., 2001; Taguchi et al., 2003). Finally, co-ligation of CD24 and the BCR with sub-optimal doses of Ab can induce apoptosis, whereas ligation of either alone cannot, suggesting cooperative signaling (Suzuki et al., 2001).

CD24 is also important in regulating T cell survival. T cells must regulate their proliferation to support a long-lived cell population, but can expand their numbers during immune activation (Boyman et al., 2009). In the absence of CD24, homeostatic proliferation of T cells is markedly reduced, however immune-driven proliferation is less affected (Li et al., 2004), likely because it depends on TCR co-receptors (Chen and Flies, 2013). When CD24+ T cells are transferred to CD24-knockout mice, excessive and destructive homeostatic T cell proliferation occurs, but CD24 expressed on dendritic cells is sufficient to ameliorate this effect (Li et al., 2006). This suggests that CD24 can act *in cis* on the T cell to regulate TCR signaling, or *in trans*, where DC-expressed CD24 can bind and modulate its partner(s) on the T cell.

#### Regulation of plasma membrane organization and signaling

As CD24 lacks an intracellular domain, it cannot directly activate intracellular signaling pathways. CD24 is resident in cholesterol-rich microdomains termed lipid rafts (Lingwood and Simons, 2010). In B cells and breast cancer cells CD24 excludes CXCR4, the receptor for Stromal Cell Derived Factor-1 (SDF-1), from lipid rafts whereas in the absence of CD24, CXCR4 can enter (Schabath et al., 2006). This exclusion prevents SDF-1-mediated CXCR4 signaling. In contrast, β-integrin is normally found in non-lipid raft membrane domains, but in the presence of CD24 it can translocate into lipid rafts (Runz et al., 2008) to promote cell-cell adhesion (Baumann et al., 2012). Therefore, these studies suggest that CD24 also regulates the physical location of surface receptors.

The ability of CD24 to act as a membrane-organizing factor further supports a role for CD24 to regulate receptor oligomerization and localization. Thus, it is possible that CD24 can rapidly or contextually alter its associations, which may be another mechanism through which it exerts context-specific effects.

## Identifying CD24 mechanisms

If CD24-mediated intracellular signaling depends on its association with cell-type specific surface receptors, identification of these partners is essential. In some cases, transcriptomic data may be used to predict partners by their co-expression with CD24 (Ayre et al., 2016), similar to the approach that identified novel ligand-receptor interactions regulating neuronal stem cell renewal (Yuzwa et al., 2016). Alternatively, identifying common signaling outcomes between CD24 and cell specific surface receptors could be used to predict receptor partners. For example, the common downstream signaling elicited by CD24 and the BCR suggests they could partner directly or via another intermediate (Suzuki et al., 2001). Visualization of co-localized receptors through high resolution microscopy may be employed to demonstrate association on intact cells, such as was used to characterize epidermal growth factor-induced receptor dimerization (Winckler et al., 2013) Alternatively, if there are no known or predicted CD24 interactors expressed in cells of interest, non-biased proteomics-based identification of CD24 interacting proteins could be used, like that used in the mass spectroscopy-based identification of N-methyl-d-aspartate receptor complexes (Husi et al., 2000).

Confirming the functional interactions between CD24 and its partner could be accomplished *in vivo* with the use of knockout and transgenic animals and *in vitro* using gene knockout or over-expression vectors, to alter the expression of CD24 and its putative signaling partner. Altering the expression of CD24 should disrupt the signaling through its partner. For example, if CD24 acts to restrict signaling, then the receptor partner may become hyper-responsive in a CD24 knockout, such as is observed with the negative regulation of the BCR by CD22 (O'Keefe et al., 1996). The inverse relationship would be seen if CD24 is a positive regulator of signaling. This relationship may explain the loss of developing B cells in both CD24 knockout and CD24-overexpressing mice, since the BCR can transduce pro-survival or pro-apoptotic signals, depending on B cell developmental status and the strength of BCR stimulation (Rajewsky, 1996; Chen et al., 1999). In CD24-knockout animals, the BCR may be over-sensitive leading to apoptosis, whereas in transgenic CD24 over-expressing mice, the BCR may no longer provide supportive tonic signaling, also leading to apoptosis.

With whole-body knockout animals, compensatory changes to the expression of the signaling partner may occur due to the absence of CD24, to re-establish their signaling potential. These changes may be observed by comparing the expression of partner receptors in wild type vs. CD24 knockout mice. The generation of inducible CD24 knock-out models, to prevent compensatory changes in partnered receptors or signaling pathways, would negate these concerns.

Importantly, knockdown or over-expression of the signaling partner would have the same biological outcomes as the loss or gain of CD24, respectively. In this case, CD24 could still be engaged with ligand or Ab, but would not exert any effect in the absence of its partner.

Determining the mechanism for CD24-ligand specificity is also key. CD24 has been shown to vary in size from approximately 30 to 80 kDa, depending on the tissue from which it is isolated due to the variable mosaic of its N- and O- linked glycosylations (Fang et al., 2010). The different terminal glycans exhibit unique binding potential to cell surface receptors. For example, Siglec-G binds to α2,6 and α2,3 sialic acid (Chen et al., 2011), however L1CAM interacts with only the α2,3 form (Bleckmann et al., 2009; Figure 1B). Contactin and TAG-1 bind to Lewis^X^ carbohydrates (Lieberoth et al., 2009) and P-selectin binds to human natural killer-1 (HNK-1) sulfated carbohydrates (Aigner et al., 1997; Figure 1B). If the binding and activity of CD24 is glycan-dependent, tissue-specific glycosylation would create glyco-variants of CD24 capable of interacting with specific partners, allow a selectivity of responsiveness, and preventing systemic effects. It is our opinion that future studies to identify *in cis* and *in trans* partners of CD24 should also identify the glycans on CD24 mediating those interactions.

## Implications and conclusions

Unlocking the CD24 signaling mechanism may have wide-ranging implications in understanding the regulation of cell fate determination in normal and cancer cells. We suggest that CD24 influences different *cis*-interacting partners, or that some CD24 “ligands” may not directly interact with CD24, but with an associated partner. This would explain how CD24 is associated with numerous ligands and cellular activities but be widely expressed and evolutionarily conserved. As CD24 is also carried on extracellular vesicles, the ability to act *in cis* or *in trans* with many partners may be significant for its functions. As a regulator of cell signaling or stress, CD24-laden vesicles may be potent signaling modulators that can interact with numerous partners in the cellular microenvironment. In our opinion, the interaction of CD24 with different signaling partners in a cell-type specific manner is the most likely explanation for the diverse effects attributed to CD24. Overall, we believe that CD24 has a single function, acting as a rheostat to modulate signaling by receptor partners and fine-tune responses to extracellular stimuli.

## Author contributions

DCA and SLC conceived the idea, and contributed equally to the preparation of the manuscript. DCA and SLC approved the final manuscript.

## Funding

Funding provided by a Discovery Grant to SLC from the Natural Sciences and Engineering Research Council of Canada (402152-2011). DCA is supported by a trainee award from the Beatrice Hunter Cancer Research Institute with funds provided by The Terry Fox Strategic Health Research Training Program in Cancer Research at CIHR and by Memorial University of Newfoundland.

### Conflict of interest statement

The authors declare that the research was conducted in the absence of any commercial or financial relationships that could be construed as a potential conflict of interest.

## Abbreviations

Ab: Antibody
BCR: B Cell Receptor
CXCR4: C-X-C chemokine receptor type 4
DAMP: Danger Associated Molecular Pattern
DC: Dendritic Cell
GPI: Glycophosphatidylinositol
HMGB1: High Mobility Group Box 1
HNK-1: Human Natural Killer-1
L1CAM: L1 Cell Adhesion Molecule
MAPK: Mitogen Activated Protein Kinase
NCAM1: Neural Cell Adhesion Molecule
PTK: Protein Tyrosine Kinase
STAT3: Signal Transducer and Activator of Transcription 3
SDF1: Stromal Cell-Derived Factor 1
TLR: Toll-Like Receptor
VEGF: Vascular Endothelial Growth Factor.
